# Evaluation of amplified rDNA restriction analysis (ARDRA) for the identification of *Mycoplasma *species

**DOI:** 10.1186/1471-2334-5-46

**Published:** 2005-06-14

**Authors:** Tim Stakenborg, Jo Vicca, Patrick Butaye, Dominiek Maes, Thierry De Baere, Rita Verhelst, Johan Peeters, Aart de Kruif, Freddy Haesebrouck, Mario Vaneechoutte

**Affiliations:** 1 Veterinary and Agrochemical Research Centre, Groeselenberg 99, 1180 Brussels, Belgium; 2 Faculty of Veterinary Medicine, Ghent University, Salisburylaan 133, 9820 Merelbeke, Belgium; 3 Department of Clinical Chemistry, Microbiology & Immunology, Ghent University Hospital, De Pintelaan 185, 9000 Ghent, Belgium

## Abstract

**Background:**

Mycoplasmas are present worldwide in a large number of animal hosts. Due to their small genome and parasitic lifestyle, *Mycoplasma *spp. require complex isolation media. Nevertheless, already over 100 different species have been identified and characterized and their number increases as more hosts are sampled. We studied the applicability of amplified rDNA restriction analysis (ARDRA) for the identification of all 116 acknowledged *Mycoplasma *species and subspecies.

**Methods:**

Based upon available 16S rDNA sequences, we calculated and compared theoretical ARDRA profiles. To check the validity of these theoretically calculated profiles, we performed ARDRA on 60 strains of 27 different species and subspecies of the genus *Mycoplasma*.

**Results:**

*In silico *digestion with the restriction endonuclease *Alu*I (AG^CT) was found to be most discriminative and generated from 3 to 13 fragments depending on the *Mycoplasma *species. Although 73 *Mycoplasma *species could be differentiated using *Alu*I, other species gave undistinguishable patterns. For these, an additional restriction digestion, typically with *Bfa*I (C^TAG) or *Hpy*F10VI (GCNNNNN^NNGC), was needed for a final identification. All *in vitro *obtained restriction profiles were in accordance with the calculated fragments based on only one 16S rDNA sequence, except for two isolates of *M. columbinum *and two isolates of the *M. mycoides *cluster, for which correct ARDRA profiles were only obtained if the sequences of both *rrn *operons were taken into account.

**Conclusion:**

Theoretically, restriction digestion of the amplified rDNA was found to enable differentiation of all described *Mycoplasma *species and this could be confirmed by application of ARDRA on a total of 27 species and subspecies.

## Background

Mycoplasmas are phylogenetically related to gram-positive bacteria with low GC-content and belong to the class of the *Mollicutes. *They form a unique group of bacteria that lack a cell-wall and that contain sterols in their cytoplasmatic membrane. They are of great importance, since several species are pathogenic to animals or humans, whereas species of other mollicute genera also infect plants and insects [1]. In addition, a series of mycoplasmas cause trouble in the laboratory, because they infect cell cultures. Already over 100 species have been described, and their number, as well as the number of different hosts is still increasing.

A correct identification of mycoplasmas, mostly performed after a fastidious initial isolation, may be achieved by various methods. Original tools to identify mycoplasmas were mainly based on biochemical and serological differentiation, varying from simple precipitation tests [2], to ELISA [3,4], immunofluorescence [5], or Western blot analysis [6]. These techniques are being replaced by faster DNA-based tools [7]. Many of these methods are based on the 16S rDNA sequence for various reasons. First, the 16S rDNA has been sequenced for all recognized *Mycoplasma *spp. and is required when describing a new species [8]. Secondly, the 16S rDNA sequences have lower intraspecific variability than most protein encoding genes, hence their use in the construction of phylogenetic topologies [9]. Recently, denaturing gradient gel electrophoresis of amplified 16S rDNA was shown to be useful to differentiate most *Mycoplasma *spp. [10]. In another approach, correct identification of related *Mycoplasma *spp. was based on differences of the 16S-23S intergenic spacer (ITS) region. Both size variation [11] as sequence differences [11,12] of the ITS were successfully used to differentiate related species. Compared to the 16S rDNA sequence, ITS sequences may vary more between strains of the same species due to a lower selection pressure [13], although reports of very highly conserved ITS regions are known as well [14].

Amplified rDNA restriction analysis (ARDRA) has already been used for the identification of some avian species [15-17] as well as for pathogenic mycoplasmas in cats [18]. Restriction analysis with *Pst*I of an amplified 16S rDNA fragment was also shown useful to differentiate *M. capricolum *subsp. *capripneumoniae *from the other species belonging to the mycoides-cluster [19]. The potential and power of ARDRA to identify members of the *Mollicutes *was already put forward [20], but was never worked out in detail for a large number of species. In this study, we investigated the value of ARDRA to identify all (to date) recognized *Mycoplasma *spp.

## Methods

### Isolates

A total of 60 strains, belonging to 27 different *Mycoplasma *species and subspecies, were used during this study (Table 1). The *Mycoplasma *spp. belonging to the mycoides-cluster and the *M. hyosynoviae *strains, were kindly provided as purified genomic DNA samples by Dr. L. Manso-Silivan (CIRAD, France) and Dr. B. Kokotovic (DFVF, Denmark), respectively. All other *Mycoplasma *spp. were cultivated using F-medium [21], modified Hayflick medium [22], SP-4-medium [22], SP-4-medium supplemented with L-arginine, HS-medium [23], or Friis'-medium with ampicillin instead of methicillin [24].

**Table 1:** List of strains used in this study

*Mycoplasma *species	Number of strains	Strain designations
*M. agalactiae*	2	NCTC 10123 (PG2); 5725
*M. arginini*	1	884/200
*M. bovigenitalium*	1	MN120
*M. bovirhinis*	3	ATCC 27748; O475; CODA 8L
*M. bovis*	4	83/61; 295VD; Widanka309; O422
*M. capricolum *subsp. *capricolum*	1	ATCC 27343 (California Kid)
*M. capricolum *subsp. *capripneumoniae*	1	NCTC 10192 (F38)
*M. columbinasale*	1	397
*M. columbinum*	4	423VD; 446; 447; 448
*M. columborale*	1	Pul46
*M. dispar*	2	ATCC 27140; MdispA
*M. flocculare*	4	ATCC 27399 (Ms42); MP102; MflocF6A; MflocF316
*M. gallinarum*	3	MgalnA; D63P; MgalnB
*M. gallisepticum*	3	ATCC 19610; A5969; 2000Myc58
*M. glycophilum*	2	412VD; MglyF1A
*M. hyopneumoniae*	4	ATCC 25934 (J); MhF56C; MhF612D; MhF72C
*M. hyorhinis*	4	MhyorF6A; MhyorF9A; MhyorF7A; MhyorF1A
*M. hyosynoviae*	4	ATCC 25591 (S16); Mp6; Mp96; Mp178
*M. lipofaciens*	1	R171
*M. mycoides *subsp. *capri*	1	Pg3
*M. mycoides *subsp. *mycoides LC*	1	YG
*M. mycoides *subsp. *mycoides SC*	1	Pg1
*M. neurolyticum*	2	MneuF1A; WVU1853
*M. orale*	1	ATCC 23714
*M. pneumoniae*	3	0696A, 1285A, 1284A
*M. putrefaciens*	4	Put85; B387; B731; 7578.95
*Mycoplasma *sp. bovine group 7	1	Pg50

All isolates were previously identified using biochemical tests and growth precipitation tests with absorbed rabbit antisera [2]. Whenever discrepancies existed between the obtained ARDRA-profiles and the serological results, the 16S rDNA was sequenced for an exact identification [25].

### DNA extraction

DNA of growing cultures was extracted using a phenol-chloroform extraction described previously [26] or using alkaline lysis. For alkaline lysis, the cultures were centrifuged (2', 10000 *g*) and resuspended in 50 μl lysis buffer (0.25% SDS in 0.05 N NaOH). After 5' at 95°C, 300 μl water was added and the bacterial debris was centrifuged (2', 10000 *g*). One μl of the supernatant was used as template for amplification of the 16S rDNA.

### 16S PCR amplification

The universal primers pA (5'AGAGTTTGATCCTGGCTCAG) and pH (5'AAGGAGGTGATCCAGCCGCA) were used to amplify the 16S rRNA genes [25], yielding an amplification product of approximately 1500 bp. Thirty cycles (20" 94°C; 15" 57°C; and 30' 72°C) were run on a GeneAmp 9600 Thermal Cycler (Perkin Elmer, USA) using 3 U recombinant *Taq *DNA polymerase (Invitrogen, UK), 1 × PCR buffer (20 mM Tris-HCl, 1.5 mM MgCl_2_, and 50 mM KCl; pH 8.4), 10 pmol of each primer and 1 μl of the genomic DNA (~30 ng) as template. Reaction volumes were 50 μl.

### Restriction digestion

For all 60 strains, 10 μl of the 16S rDNA PCR product was digested with 5 U of restriction enzyme *Alu*I (Fermentas, Lithuania; sequence: AG^CT) and the associated Y^+^/Tango restriction buffer (Fermentas) in a total volume of 20 μl for 2 hours at 37°C. For a final identification, the amplified 16S rDNA of some strains were digested in addition with *Bfa*I (New England Biolabs, USA; sequence: C^TAG) or *Hpy*F10VI (Fermentas; sequence: GCNNNNN^NNGC). The restriction fragments were separated on a 3% Nusieve 3:1 agar (Tebu-Bio, France) for 2 hours at 130 V and visualized using a GeneGenius gel documentation system (Westburg, The Netherlands). A 50-bp ladder was used as a DNA marker (Fermentas).

### Sequences &*in silico *ARDRA-profiles

**Figure 1:**
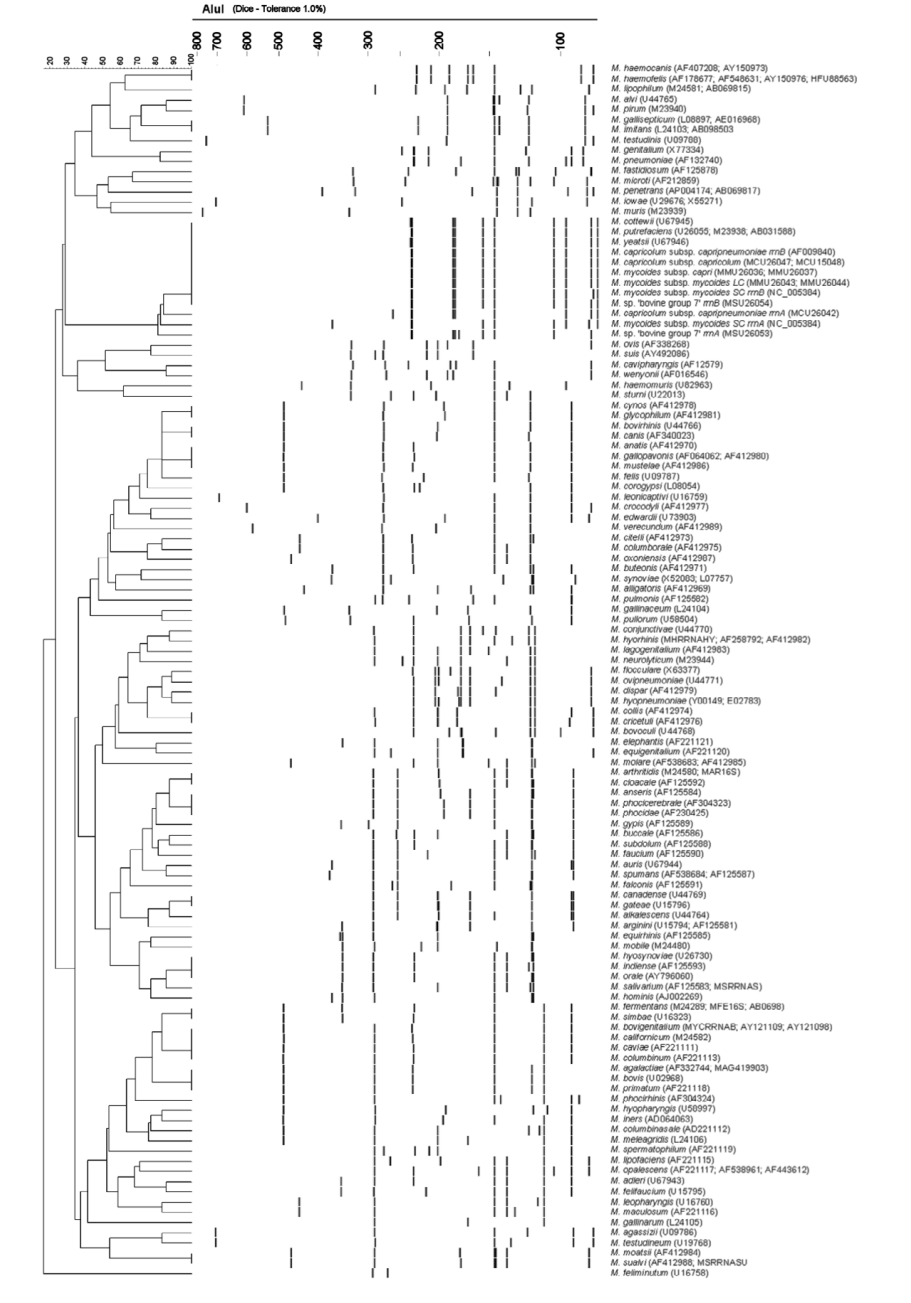
Theoretical ARDRA patterns after *in silico *digestion with *Alu*I for all currently recognized *Mycoplasma *spp. Patterns are clustered using UPGMA (Bionumerics V3.5) by way of illustration. The Genbank-accession numbers used are listed together with species name.

ARDRA-profiles were calculated for all *Mycoplasma *spp. as acknowledged by the International Committee on Systematics of Prokaryotes (ICPS) to date. The 16S rDNA sequences were downloaded from Genbank (accession numbers are indicated in Figure 1). A consensus sequence was constructed and used for species for which more than one sequence was available. The *M. orale *16S rDNA sequence was determined and submitted [Genbank:AY796060], since the only available sequence contained numerous ambiguities. For the members of the *M. mycoides*-cluster – for which differences between *rrn*A and *rrn*B have been published [27] – both sequences were used. For some *Mycoplasma *spp. only a partial sequence of the 16S rDNA was available. For these sequences, nucleotides were added to the 5' and/or 3' ends to generate fragments of expected length. These lengths and the choice of the nucleotides added were based on a 16S rDNA consensus sequence obtained by alignment of the complete *Mycoplasma *16S rDNA sequences available in Genbank using Clustal W. The restriction sites and the exact size of the ARDRA fragments were calculated using Vector NTI Advance V9.0 (Invitrogen) and BioNumerics V3.5 (Applied-Maths, Belgium).

By way of illustration, a dendrogram, based on ARDRA patterns, was constructed using the Unweighted Pair Group Method with Arithmetic Means (UPGMA) using 1% tolerance (*i.e. *bands that differ about 7 nucleotides or less are considered identical) and taking only fragments from 80 to 800 nucleotides into account.

## Results

**Figure 2:**
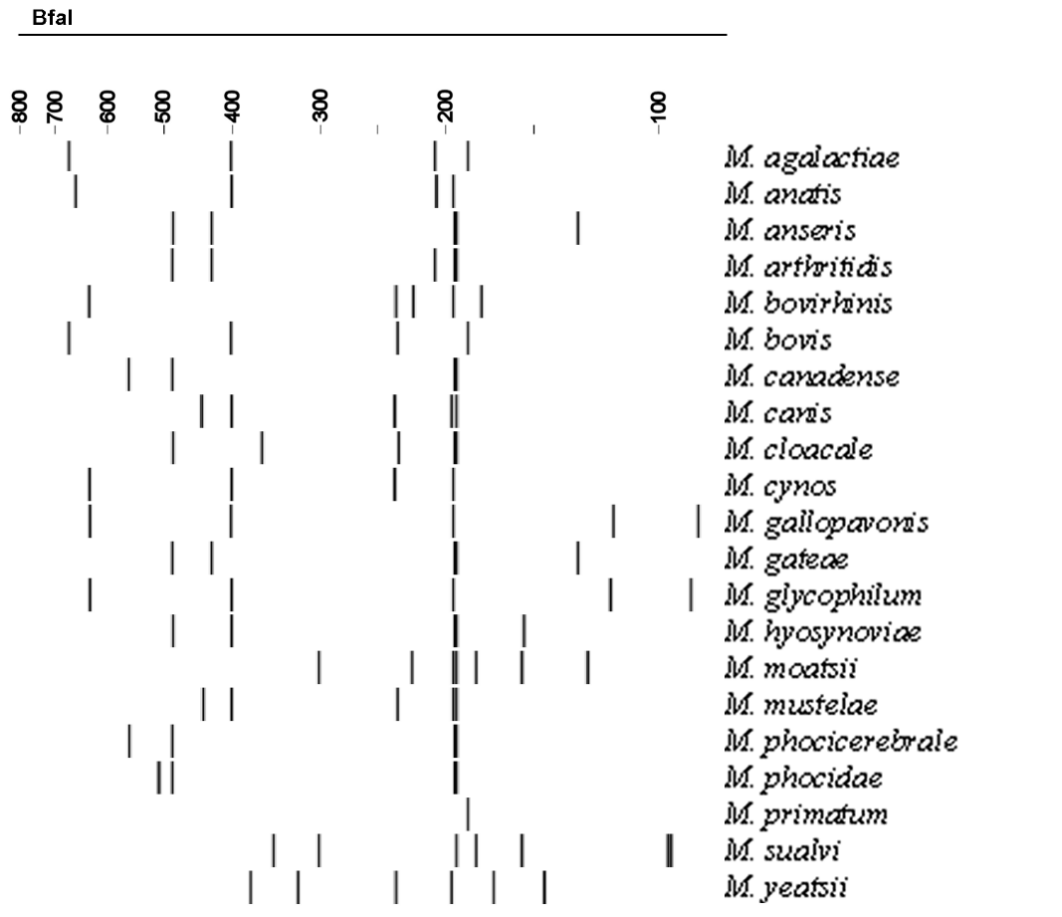
Calculated ARDRA profiles of *Mycoplasma *spp. that can be differentiated using *Bfa*I, but had undistinguishable *Alu*I restriction profiles.

For all *Mycoplasma *spp., the theoretical *Alu*I,*Bfa*I and *Hpy*F10VI restriction patterns were calculated [see Additional file 1] and are represented in Figure 1, 2, 3. For a number of species, ARDRA was carried out in the laboratory to confirm the *in silico *obtained results and to check the validity of the technique for identification. ARDRA profiles obtained with *Alu*I and *Bfa*I are shown in Figure 4 and Figure 5, respectively. For a further verification of the technique and for the remaining 9 species that could not be identified with *Alu*I or *Bfa*I alone, ARDRA was also performed with *Hpy*F10VI (Figure 6, 7).

**Figure 3:**
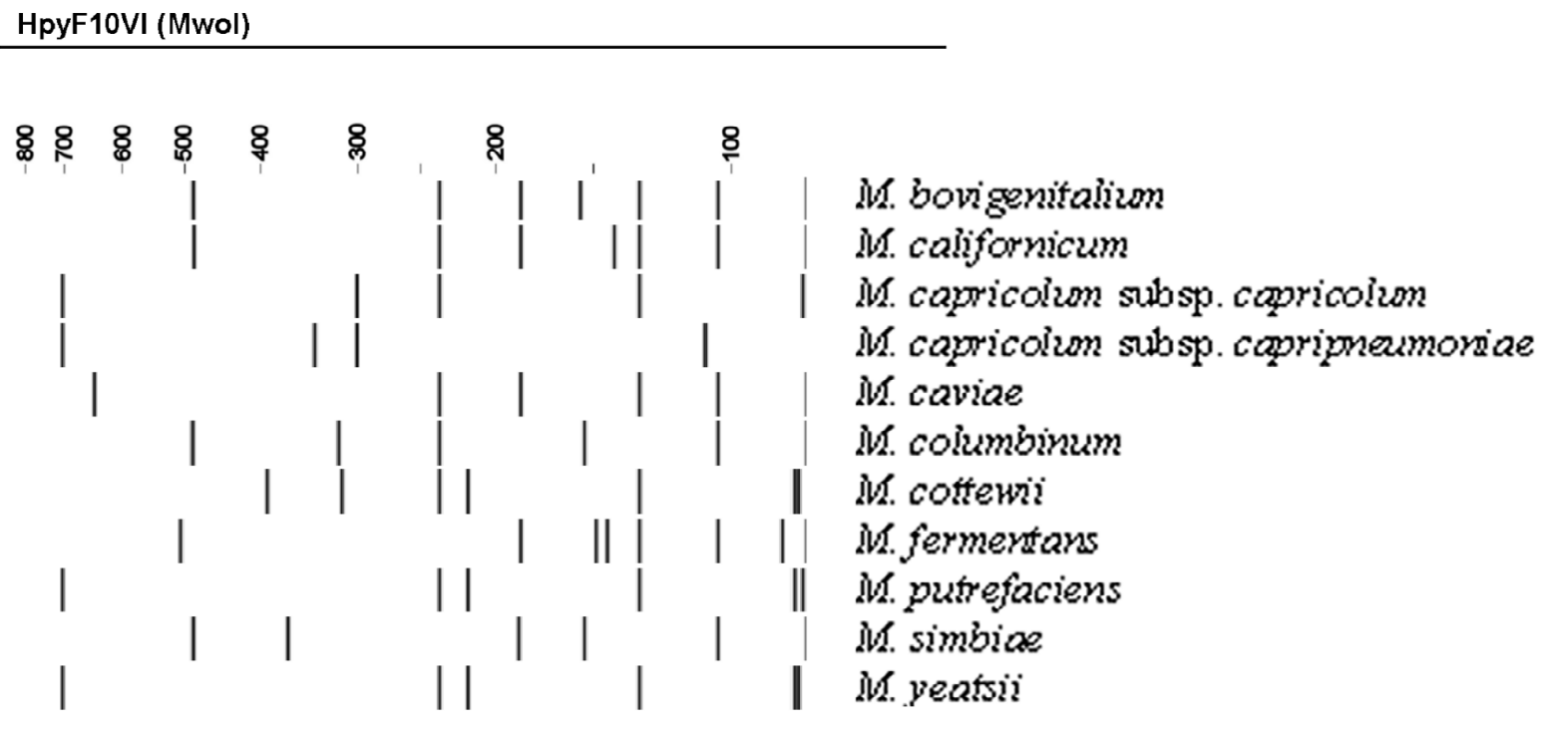
Calculated ARDRA profiles of *Mycoplasma *spp. that can be differentiated using *Hpy*F10VI, but had undistinguishable *Alu*I restriction profiles. The restriction pattern of *M. capricolum *subsp. *capricolum *represents the not included members of the *M. mycoides*-cluster as well.

**Figure 4:**
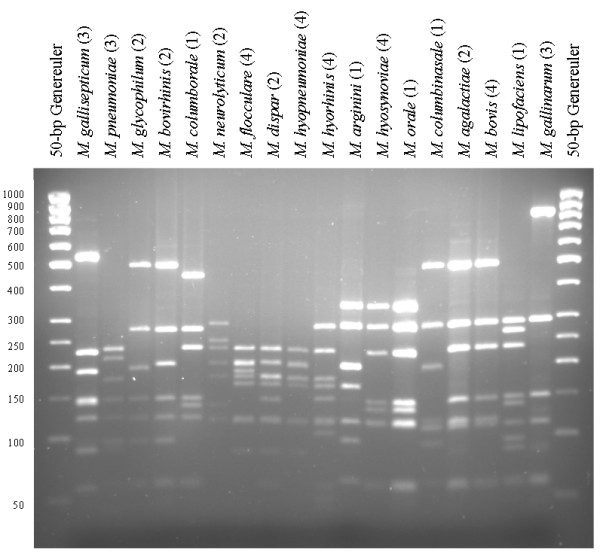
ARDRA profiles after restriction with *Alu*I of 18 different *Mycoplasma *species. Since all samples of the same species gave identical restriction patterns, the number of strains tested for each species is indicated in parenthesis. A Generuler 50-bp ladder (Fermentas) was used as size-marker.

**Figure 5:**
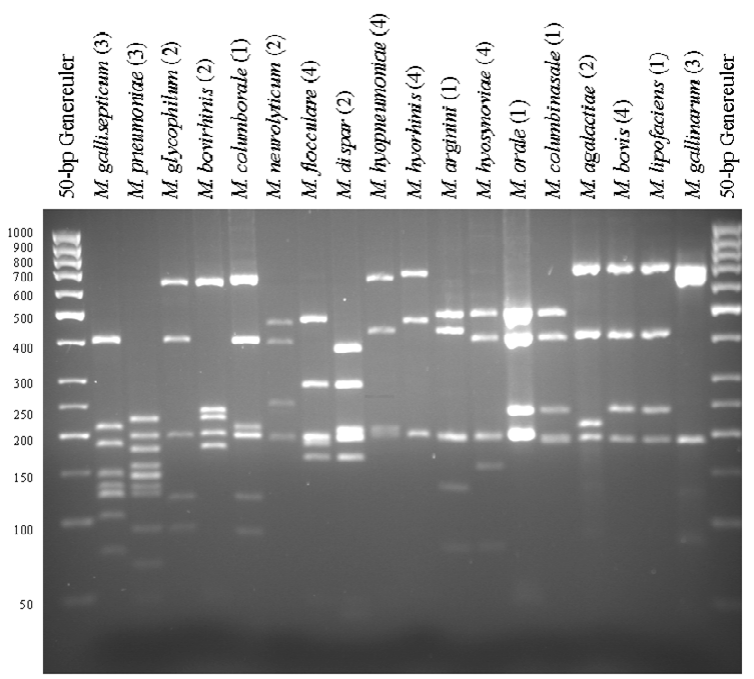
ARDRA profiles after restriction with *Bfa*I of 18 different *Mycoplasma *species. Since all samples of the same species gave identical restriction patterns, the number of strains tested for each species is indicated in parenthesis. A Generuler 50-bp ladder (Fermentas) was used as size-marker.

**Figure 6:**
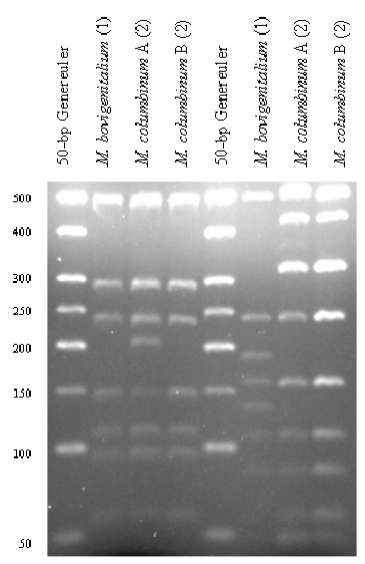
ARDRA profiles after restriction with *Alu*I (left) or *Hpy*F10VI (right) of *M. bovigenitalium *and of *M. columbinum*. A Generuler 50-bp ladder (Fermentas) was used as size-marker. The number of strains tested for each species is indicated in parenthesis.

**Figure 7:**
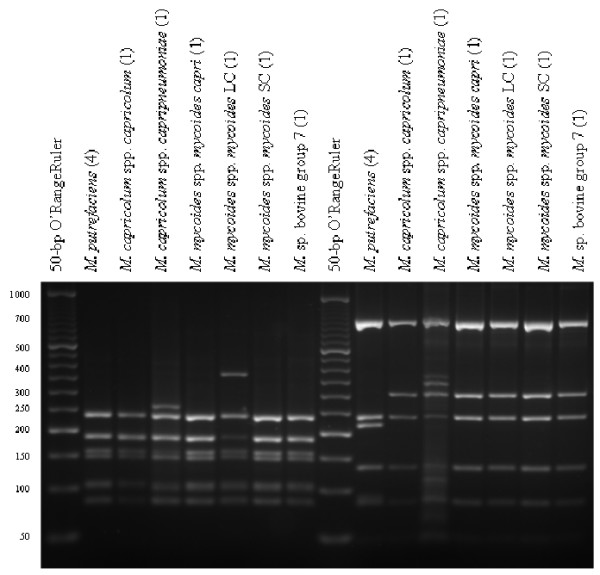
ARDRA profiles of *M. putrefaciens *and the *M. mycoides *cluster after restriction with *Alu*I (left) and *Hpy*F10VI (right). The expected band sizes for both *rrn *operons are indicated in Additional file 1. An O'RangeRuler 50-bp ladder (Fermentas) was used as size-marker. The number of strains tested for each species is indicated in parenthesis.

Two of the four *M. columbinum *strains showed an unpredicted ARDRA pattern after restriction with *Alu*I. Since the sum of all bands was higher than the length of the 16S sequence, a difference between the 2 *rrn *operons was expected. This was verified by sequence analysis, which revealed an ambiguity at position 997 (*i.e. *position 1007 in the *E. coli *numbering), pointing to the presence of AGCT in one and AGTT in the other operon. As such, a restriction site for *Alu*I in one operon will lack in the other operon and will lead to a mixture of ARDRA profiles. Also for the strains of the *M. mycoides*-cluster the published sequences of both *rrn *operons were taken into account [27]. By superimposition of the restriction profiles of both *rrn*A and *rrn*B, the correct, expected profiles were obtained. However, a faint band of approximately 370 nucleotides was observed in the *Hpy*F10VI restriction profile of *M. capricolum *subsp. *capripneumoniae*, indicating a partial restriction at position 1082 of the *rrnA *gene (Figure 7). For all other samples, profiles were identical to the calculated restriction profiles using only one consensus sequence of the Genbank entries.

A few species could not be differentiated with the three suggested enzymes and for these, other enzymes were selected. *M. cricetuli *and *M. collis*, which have 16S rRNA operons that are 99.8% identical, can be differentiated using *Hpy*188III. This enzyme cuts the 16S rDNA of *M. collis *7 times, while restriction takes place only 6 times in the 16S rRNA gene of *M. cricetuli*. Also the restriction enzyme *Ear*I can be used, since it only restricts the 16S rRNA gene of *M. cricetuli*. The very related *M. imitans *and *M. gallisepticum *could be differentiated using *Mse*I or *Hind*II. The restriction enzyme *Bst*UI could be used to differentiate the otherwise indistinguishable *M. haemocanis *(2 restriction sites) and *M. haemofelis *(3 restriction sites). The determined 16S rDNA sequence of *M. orale *was almost identical to the 16S rDNA of *M. indiense *and specific restriction enzymes, like *Bsa*JI or *Eco*HI, were necessary to differentiate these species. In case of the very related members of the mycoides-cluster, the differentiation is more complicated and a whole series of restrictions are needed. Based on the occurrence of different restriction sites, it is however theoretically possible to correctly identify these species as well, using only commercially available restriction endonucleases (Table 2).

**Table 2:** Number of restriction sites for the members of the *M. mycoides*-cluster

	*Mycoplasma *species					
	
Restriction endonuclease	*Mycoplasma *sp. bovine group 7	*M. mycoides *ssp. *mycoides *LC	*M. mycoides *ssp. *capri*	*M. mycoides *ssp. *mycoides *SC	*M. capricolum *ssp. *capripneumoniae*	*M. capricolum *ssp. *capricolum*
*Bbv*I	4	4	4	4	4/2	4
*Hpy*CH4III	3	4	4	3	3	3
*Hpy*F10VI	5	5	5	5	5/4	5
*Mae*III	5	5	5	4	5	5
*Mbo*II	3/5^a^	3	3	3	3	3/4
*Tsp*509I	4	4	4	4/5	4	4

^a ^Two values indicate differences between *rrn*A and *rrn*B, based on the Genbank accession numbers indicated in 1.

## Discussion

Identification of mycoplasmas still largely relies on serological tests, but owing to the limited availability of quality-controlled sera, the high number of species, the serological cross-reaction between related species and the great variability in the surface antigens of different strains [28], newer techniques are needed. Sequence analysis of the 16S rRNA genes proved a useful tool to identify species, but the need for expensive equipment makes the technique less favorable for routine diagnosis. In this study, we showed that theoretically all *Mycoplasma *spp. are distinguishable using ARDRA. The *in silico *determined discriminative power was confirmed in the laboratory and even closely related *Mycoplasma *spp. could be identified correctly, as exemplified by the restriction with *Alu*I and *Bfa*I of *M. agalactiae *and *M. bovis*.

We used universal primers to amplify the entire 16S rDNA to obtain a maximum discriminatory power. Working with universal primers implies that interference from other bacteria is to be expected when starting from clinical samples [29], especially when mycoplasmas are not abundantly present. The use of mycoplasma-specific primers binding to internal regions of the 16S rRNA genes may be helpful and result in a higher specificity as was already proposed by others [20,30]. However, care must be taken since the discriminatory power will decrease if primers are chosen in such a way that less restriction sites are present in the amplification products. Alternatively, McAuliffe *et al*. [31] proposed a selective enrichment step for 24 hours in Eaton's-medium before amplification of 16S sequences to identify *Mycoplasma *spp. Also Kiss *et al*. [16] used ARDRA to identify three avian *Mycoplasma *species after 48 hours of incubation in Frey media. These suggested approaches may solve most problems, but may still be insufficient for mixed *Mycoplasma *cultures. The presence of more than one *Mycoplasma *species in clinical samples will lead to complex patterns, which are not easily resolved.

Differences between *rrn *operons have been reported in several bacterial classes, but the level of sequence heterogeneity was recently shown to be lower than expected [32]. It is therefore reasonable to assume that *rrn *operons tend to evolve in concert [33]. For some bacterial species a high level of 16S rDNA sequence heterogeneity has been described [34,35], while for *Mycoplasma *species, which possess no more than 2 *rrn *operons, only some micro-heterogeneity (*i.e. *scattered sequence variation between highly related rRNA genes) has been reported [27,36,37]. Besides, most differences between the two operons will not lead to altered restriction sites and will not influence the ARDRA patterns. In case a mutation is located within one of both restriction recognition sites, as was shown in particular for *M. columbinum*, restriction will most likely yield an unknown ARDRA profile, rather than lead to a false identification. Moreover, this aberrant pattern can be included in the identification scheme. The significance of the C1007T transition (*E. coli *numbering) present in two of the four *M. columbinum *strains is still unknown, but was shown in some strains of *E. coli *as well [33]. Also, in agreement with an earlier report [27], many differences between the *rrn*A and *rrn*B sequences were observed for members of the *M. mycoides *cluster. Nevertheless, the combined restriction profiles of both *rrn *sequences resulted in expected patterns with exception of a faint band seen for *M. capricolum *subsp. *capripneumoniae *after restriction with *Hpy*F10VI. The reason for this partial restriction is unknown since purifying the PCR product, increasing the enzyme concentration, or lengthening the incubation period made no difference (data not shown). In any case, identification based on ARDRA was shown complex for these very related species and other techniques – like serological tests independent of the 16S rDNA sequences [8] – may be more suitable. However, the extra band visible for *M. mycoides *subsp. *mycoides *SC after restriction with *Alu*I was shown sufficiently stable to be used for identification [38] and the value of ARDRA using *Pst*I was also reported for *M. capricolum *subsp. *capripneumoniae *[36]. Although the 16S rDNA sequences of these species may be almost identical, ARDRA is able to emphasize the few differences present without the need of extensive 16S rDNA sequence analysis or other tests [19,38-40]. Also for other species with nearly identical 16S rDNA sequences (99.5% identity for *M. haemocanis *and *M. haemofelis*; 99.7% for *M. gallisepticum *and *M. imitans*; 98.9% for *M. orale *and *M. indiense*, and 99.8% for *M. criteculi *and *M. collis*), it was calculated that restriction analysis with a single additional enzyme would result in different restriction patterns and therefore to a correct identification.

## Conclusion

Restriction digestion with *Alu*I of the amplified 16S rDNA can be used to differentiate between 73 of the 116 described *Mycoplasma *species and subspecies. An additional restriction with *Bfa*I or *Hpy*F10VI enables the identification of another 31 species and subspecies. Also the remaining 12 species can be differentiated, with the use of additonal enzymes, although other techniques may be preferred for some members of the *M. mycoides*-cluster.

The simplicity and the general applicability of ARDRA make it possible to implement this technique in most laboratories with basic molecular biology equipment.

## List of abbreviations

ARDRA amplified rDNA restriction analysis

ITS intergenic spacer(s)

CIRAD Agricultural Research Centre for International Development (Montpellier, France)

DFVF Danish Institute for Food and Veterinary Research (Copenhagen, Denmark)

UPGMA Unweighted Pair Group Method with Arithmatic Means

## Competing interests

The author(s) declare that they have no competing interests.

## Authors' contributions

TS collected and analyzed most of the data and was principal writer of the manuscript. TDB participated in the initial *in silico *data analysis, while RV helped in the correct identification of the species. JV, PB, DM, JP, and AdK co-drafted the manuscript. FH participated in the discussion of the data, participated in proofreading and management. MV conceived the study and revised the manuscript critically. All authors made contributions, read and approved the final manuscript.

## Pre-publication history

The pre-publication history for this paper can be accessed here:



## Supplementary Material

Additional File 1 **Overview of the restriction fragments (and corresponding restriction sites) after ARDRA with *Alu*I, *Bfa*I and *Hpy*F10VI for all current 116 *Mycoplasma *species and subspecies. The restriction enzymes needed to obtain a correct identification are marked in bold. The fragments are listed according to their size**. Based upon available 16S rDNA sequences, ARDRA profiles were calculated for *Alu*I, *Bfa*I and *Hpy*F10VI for all currently acknowledged *Mycoplasma *species. For these restriction enzymes, the file gives a detailed overview of the in silico determined restriction sites as well as the size of the restriction fragments. Click here for file
