# Supplementary material for: Evaluation of amplified rDNA restriction analysis (ARDRA) for the identification of Mycoplasma species
Source: BMC Infect Dis. 2005 Jun 14;5:46. doi: 10.1186/1471-2334-5-46 (PMC1177949; doi:10.1186/1471-2334-5-46)
Supplement: Additional File 1 — Overview of the restriction fragments (and corresponding restriction sites) after ARDRA with AluI, BfaI and HpyF10VI for all current 116 Mycoplasma species and subspecies. The restriction enzymes needed to obtain a correct identification are marked in bold. The fragments are listed according to their size. Based upon available 16S rDNA sequences, ARDRA profiles were calculated for AluI, BfaI and HpyF10VI for all currently acknowledged Mycoplasma species. For these restriction enzymes, the file gives a detailed overview of the in silico determined restriction sites as well as the size of the restriction fragments. [file 1471-2334-5-46-S1.doc]

Additional File 1: Overview of the restriction fragments (and corresponding restriction sites) after ARDRA with *Alu*I, *Bfa*I and *Hpy*F10VI for all current 116 *Mycoplasma* species and subspecies^1^. The restriction enzymes needed to obtain a correct identification are marked in bold^2^. The fragments are listed according to their size.

| *Mycoplasma* spp. | Restriction endonucleases | | |
| --- | --- | --- | --- |
|  | AluI | BfaI | HpyF10VI (MwoI) |
| *M. adleri* | **352 (370-721), 291 (1044-1334), 232 (1-232), 147 (841-987), 137 (233-369), 112 (1394-1505), 95 (722-816), 59 (1335-1393), 56 (988-1043), 24 (817-840)** | 681 (637-1317), 403 (234-636), 233 (1-233), 188 (1318-1505) | 489 (230-718), 237 (844-1080), 187 (1213-1399), 164 (66-229), 132 (1081-1212), 106 (1400-1505), 81 (763-843), 56 (1-56), 44 (719-762), 9 (57-65) |
| *M. agalactiae* | **489 (234-722), 291 (1045-1335), 233 (1-233), 147 (842-988), 119 (723-841), 112 (1395-1506), 59 (1336-1394), 56 (989-1044),** | **681 (638-1318), 403 (235-637), 207 (1-207), 188 (1319-1506), 27 (208-234)** | 489 (231-719), 319 (1082-1400), 237 (845-1081), 165 (66-230), 106 (1401-1506), 81 (764-844), 56 (1-56), 35 (729-763), 9 (57-65), 9 (720-728) |
| *M. agassizii* | **720 (1-720), 291 (1046-1336), 147 (843-989), 122 (721-842), 95 (1421-1515), 84 (1337-1420), 56(990-1045)** | 682 (638-1319), 564 (74-637), 196 (1320-1515), 65 (1-65), 8 (66-73) | 525 (193-717), 237 (846-1082), 186 (1216-1401), 136 (57-192), 133 (1083-1215), 114 (1402-1515), 78 (762-839), 56 (1-56), 44 (718-761), 6;  840-845) |
| *M. alkalescens* | **293 (1042-1334), 255 (465-719), 201 (1-201), 169 (202-370), 147 (839-985), 119 (720-838), 96 (1418-1513), 94 (371-464), 59 (1335-1393), 56 (986-1041), 24 (1394-1417)** | 487 (637-1123), 429 (208-636), 196 (1318-1513), 194 (1124-1317), 132 (76-207), 75 (1-75) | 518 (199-716), 435 (1079-1513), 237 (842-1078), 101 (57-157), 81 (761-841), 56 (1-56), 44 (717-760), 41 (158-198) |
| *M. alligatoris* | **435 (168-602), 277 (1041-1317), 203 (838-1040), 167 (1-167), 121 (1377-1497), 118 (603-720), 95 (721-815), 59 (1318-1376), 22 (816-837)** | 501 (135-635), 471 (636-1106), 197 (1301-1497), 194 (1107-1300), 134 (1-134) | 568 (150-717), 305 (1078-1382), 237 (841-1077), 115 (1383-1497), 79 (762-840), 56 (1-56), 53 (57-109), 40 (110-149), 35 (727-761), 9 (718-726) |
| *M. alvi* | **612 (235-846), 192 (1051-1242), 148 (847-994), 146 (1-146), 143 (1243-1385), 122 (1386-1507), 88 (147-234), 56 (995-1050)** | 399 (236-634), 186 (786-971), 146 (640-785), 145 (972-1116), 126 (1184-1309), 123 (1310-1432), 104 (132-235), 76 (1-76), 75 (1433-1507), 67 (1117-1183), 55 (77-131), 5 (635-639) | 669 (181-849), 302 (1206-1507), 238 (850-1087), 152 (1-152), 118 (1088-1205), 28 (153-180) |
| *M. anatis* | **277 (1043-1319), 232 (1-232), 147 (840-986), 121 (1379-1499), 95 (721-815), 59 (1320-1378), 56 (987-1042), 24 (816-839)** | **402 (234-635), 206 (1-206), 197 (1303-1499), 27 (207-233)** | 661 (57-717), 318 (762-1079), 305 (1080-1384), 115 (1385-1499), 56 (1-56), 35 (727-761), 9 (718-726) |
| *M. anseris* | **293 (1039-1331), 255 (463-717), 199 (1-199), 169 (200-368), 147 (836-982), 120 (1391-1510), 118 (718-835), 94 (369-462), 59 (1332-1390), 56 (983-1038)** | **429 (206-634), 196 (1315-1510), 194 (1121-1314), 131 (75-205), 74 (1-74)** | 658 (57-714), 435 (1076-1510), 237 (839-1075), 80 (759-838), 56 (1-56), 44 (715-758) |
| *M. arginini* | **350 (373-722), 293 (1045-1337), 203 (1-203), 203 (842-1044), 169 (204-372), 119 (723-841), 95 (1421-1515), 59 (1338-1396), 24 (1397-1420)** | 487 (640-1126), 430 (210-639), 195 (1321-1515), 194 (1127-1320), 132 (78-209), 77 (1-77) | 519 (201-719), 434 (1082-1515), 237 (845-1081), 102 (58-159), 81 (764-844), 57 (1-57), 44 (720-763), 41 (160-200) |
| *M. arthritidis* | **293 (1042-1334), 255 (465-719), 201 (1-201), 137 (234-370), 120 (1394-1513), 119 (720-838), 105 (839-943), 94 (371-464), 59 (1335-1393), 56 (986-1041), 42 (944-985), 32 (202-233)** | **487 (637-1123), 391 (208-598), 207 (1-207), 196 (1318-1513), 194 (1124-1317), 38 (599-636)** | 558 (159-716), 435 (1079-1513), 237 (842-1078), 93 (66-158), 81 (761-841), 56 (1-56), 44 (717-760), 9 (57-65) |
| *M. auris* | **370 (1-370), 293 (1042-1334), 255 (465-719), 147 (839-985), 119 (720-838), 96 (1418-1513), 94 (371-464), 59 (1335-1393), 56 (986-1041), 24 (1394-1417)** | 561 (76-636), 487 (637-1123), 196 (1318-1513), 194 (1124-1317), 75 (1-75) | 518 (199-716), 435 (1079-1513), 237 (842-1078), 101 (57-157), 81 (761-841), 56 (1-56), 44 (717-760), 41 (158-198) |
| *M. bovigenitalium* | **489 (235-723), 291 (1046-1336), 234 (1-234), 147 (843-989), 112 (1396-1507), 95 (724-818), 59 (1337-1395), 56 (990-1045), 24 (819-842)** | 487 (639-1125), 403 (236-638), 235 (1-235), 194 (1126-1319), 188 (1320-1507) | **489 (232-720), 237 (846-1082), 187 (1215-1401), 157 (57-213), 132 (1083-1214), 106 (1402-1507), 81 (765-845), 56 (1-56), 44 (721-764), 9 (214-222), 9 (223-231)** |
| *M. bovirhinis* | **488 (235-722), 276 (1046-1321), 202 (1-202), 147 (843-989), 121 (1381-1501), 95 (723-817), 59 (1322-1380), 56 (990-1045), 32 (203-234), 25 (818-842)** | **638 (667-1304), 235 (1-235), 223 (236-458), 197 (1305-1501), 179 (459-637), 29 (638-666)** | 663 (57-719), 304 (1083-1386), 237 (846-1082), 115 (1387-1501), 82 (764-845), 56 (1-56), 35 (729-763), 9 (720-728) |
| *M. bovis* | **489 (234-722), 291 (1045-1335), 233 (1-233), 147 (842-988), 119 (723-841), 112 (1395-1506), 59 (1336-1394), 56 (989-1044)** | **681 (638-1318), 403 (235-637), 234 (1-234), 188 (1319-1506)** | 489 (231-719), 319 (1082-1400), 237 (845-1081), 165 (66-230), 106 (1401-1506), 81 (764-844), 56 (1-56), 35 (729-763), 9 (57-65), 9 (720-728) |
| *M. bovoculi* | **233 (382-614), 190 (1155-1344), 179 (1345-1523), 177 (205-381), 146 (852-997), 120 (732-851), 117 (615-731), 101 (1054-1154), 84 (73-156), 72 (1-72), 56 (998-1053), 48 (157-204)** | 457 (220-676), 290 (844-1133), 219 (1-219), 196 (1328-1523), 194 (1134-1327), 167(677-843) | 728 (1-728), 318 (773-1090), 301 (1223-1523), 132 (1091-1222), 44 (729-772) |
| *M. buccale* | **293 (1041-1333), 257 (463-719), 231 (1-231), 203 (838-1040), 137 (232-368), 120 (1393-1512), 118 (720-837), 94 (369-462), 59 (1334-1392)** | 634 (1-634), 488 (635-1122), 196 (1317-1512), 194(1123-1316) | 558 (159-716), 435 (1078-1512), 237 (841-1077), 93 (66-158), 80 (761-840), 56 (1-56), 44 (717-760), 9 (57-65) |
| *M. buteonis* | **370 (233-602), 277 (1041-1317), 232 (1-232), 203 (838-1040), 121 (1377-1497), 118 (603-720), 95 (721-815), 59 (1318-1376), 22 (816-837)** | 442 (665-1106), 402 (234-635), 206 (1-206), 197 (1301-1497), 194 (1107-1300), 29 (636-664), 27(207-233) | 661 (57-717), 420 (1078-1497), 237 (841-1077), 79 (762-840), 56 (1-56), 35 (727-761), 9 (718-726) |
| *M. californicum* | **488 (234-721), 291 (1044-1334), 233 (1-233), 147 (841-987), 112 (1394-1505), 95 (722-816), 59 (1335-1393), 56 (988-1043), 24 (817-840)** | 681 (637-1317), 402 (235-636), 234 (1-234), 188(1318-1505) | **488 (231-718), 237 (844-1080), 187 (1213-1399), 142 (57-198), 132 (1081-1212), 106 (1400-1505), 81 (763-843), 56 (1-56), 44 (719-762), 14 (199-212), 9 (213-221), 9 (222-230)** |
| *M. canadense* | **293 (1043-1335), 255 (466-720), 203 (840-1042), 202 (1-202), 169 (203-371), 119 (721-839), 96 (1419-1514), 94 (372-465), 59 (1336-1394), 24 (1395-1418)** | **561 (77-637), 487 (638-1124), 196 (1319-1514), 194 (1125-1318), 76 (1-76)** | 518 (200-717), 435 (1080-1514), 237 (843-1079), 102 (57-158), 81 (762-842), 56 (1-56), 44 (718-761), 41 (159-199) |
| *M. canis* | **488 (236-723), 276 (1046-1321), 203 (1-203), 147 (843-989), 122 (1381-1502), 95 (724-818), 59 (1322-1380), 56 (990-1045), 32 (204-235), 24 (819-842)** | **443 (668-1110), 402 (237-638), 236 (1-236), 198 (1305-1502), 194 (1111-1304), 29 (639-667)** | 520 (201-720), 318 (765-1082), 304 (1083-1386), 144 (57-200), 116 (1387-1502), 56 (1-56), 35 (730-764), 9 (721-729) |
| *M. capricolum* ssp. *capricolum* | 236 (605-840), 234 (1-234), 186 (235-420), 184 (421-604), 157 (988-1144), 147 (841-987), 105 (1145-1249), 99 (1417-1515), 85 (1250-1334), 82(1335-1416) | 378 (260-637), 352 (784-1135), 235 (1-235), 172 (1146-1317), 146 (638-783), 134 (1318-1451), 64 (1452-1515), 24 (236-259), 10 (1136-1145) | 717 (1-717), 303 (1213-1515), 237 (844-1080), 132 (1081-1212), 82 (762-843), 44 (718-761) |
| *M. capricolum* ssp. *capripneumoniae* | **262 (988-1249)^b^, 236 (605-840), 234 (1-234) , 186 (235-420), 184 (421-604), 157 (988-1144)^a^, 147 (841-987), 105 (1145-1249)^a^, 99 (1417-1515), 85 (1250-1334), 82 (1335-1416)** | 378 (260-637), 352 (784-1135), 235 (1-235), 182 (1136-1317)^b^, 172 (1146-1317)^a^, 146 (638-783), 134 (1318-1451), 64 (1452-1515), 24 (236-259), 10 (1136-1145)^a^ | **717 (1-717), 342 (871-1212)^b^, 303 (1213-1515), 237 (844-1080)^a^, 132 (1081-1212)^a^, 109 (762-870)^b^, 82 (762-843)^a^, 44 (718-761)** |
| *M. caviae* | **489 (233-721), 291 (1044-1334), 232 (1-232), 147 (841-987), 112 (1394-1505), 95 (722-816), 59 (1335-1393), 56 (988-1043), 24 (817-840)** | 681 (637-1317), 403 (234-636), 233 (1-233), 188 (1318-1505) | **653 (66-718), 237 (844-1080), 187 (1213-1399), 132 (1081-1212), 106 (1400-1505), 81 (763-843), 56 (1-56), 44 (719-762), 9 (57-65)** |
| *M. cavipharyngis* | **329 (274-602), 273 (1-273), 240 (603-842), 189 (1131-1319), 184 (1320-1503), 147 (843-989), 85 (1046-1130), 56 (990-1045)** | 635 (1-635), 479 (824-1302), 146 (636-781), 126 (1303-1428), 75 (1429-1503), 42 (782-823) | 845 (1-845), 421 (1083-1503), 237 (846-1082) |
| *M. citelli* | **446 (372-817), 277 (1043-1319), 234 (1-234), 147 (840-986), 121 (1379-1499), 118 (254-371), 59 (1320-1378), 56 (987-1042), 22 (818-839), 19 (235-253)** | 484 (638-1121), 402 (236-637), 235 (1-235), 197 (1303-1499), 181 (1122-1302) | 707 (57-763), 305 (1080-1384), 237 (843-1079), 115 (1385-1499), 79 (764-842), 56 (1-56) |
| *M. cloacale* | **293 (1040-1332), 255 (464-718), 200 (1-200), 147 (837-983), 137 (233-369), 120 (1392-1511), 118 (719-836), 94 (370-463), 59 (1333-1391), 56 (984-1039), 32 (201-232)** | **486 (636-1121), 364 (234-597), 233 (1-233), 196 (1316-1511), 194 (1122-1315), 38 (598-635)** | 518 (198-715), 435 (1077-1511), 237 (840-1076), 141 (57-197), 74 (760-833), 56 (1-56), 44 (716-759), 6 (834-839) |
| *M. collis* | 291 (1058-1348), 233 (385-617), 203 (855-1057), 202 (1-202), 182 (203-384), 120 (735-854), 117 (618-734), 96 (1433-1528), 84 (1349-1432) | 679 (1-679), 458 (680-1137), 197 (1332-1528), 194 (1138-1331), | 731 (1-731), 434 (1095-1528), 237 (858-1094), 82 (776-857), 44 (732-775) |
| *M. columbinasale* | **489 (235-723), 291 (1046-1336), 203 (843-1045), 120 (1-120), 114 (121-234), 112 (1396-1507), 95 (724-818), 59 (1337-1395), 24 (819-842)** | 487 (639-1125), 403 (236-638), 235 (1-235), 194 (1126-1319), 188 (1320-1507) | 489 (232-720), 319 (1083-1401), 237 (846-1082), 175 (57-231), 106 (1402-1507), 81 (765-845), 56 (1-56), 44 (721-764) |
| *M. columbinum* | **490 (233-722), 291 (1045-1335), 232 (1-232), 147 (842-988), 112 (1395-1506), 95 (723-817), 59 (1336-1394), 56 (989-1044), 24 (818-841)** | 681 (638-1318), 404 (234-637), 233 (1-233), 188 (1319-1506) | **490 (230-719), 319 (1082-1400), 237 (845-1081), 155 (57-211), 106 (1401-1506), 81 (764-844), 56 (1-56), 44 (720-763), 9 (212-220), 9 (221-229)** |
| *M. columborale* | **446 (371-816), 277 (1042-1318), 233 (1-233), 147 (839-985), 137 (234-370), 121 (1378-1498), 59 (1319-1377), 56 (986-1041), 22 (817-838)** | 665 (637-1301), 402 (235-636), 197 (1302-1498), 118 (1-118), 89 (119-207), 27 (208-234) | 706 (57-762), 305 (1079-1383), 237 (842-1078), 115 (1384-1498), 79 (763-841), 56 (1-56) |
| *M. conjunctivae* | **292 (1056-1347), 233 (383-615), 179 (1348-1526), 169 (214-382), 156 (1-156), 146 (854-999), 121 (733-853), 117 (616-732), 57 (157-213), 56 (1000-1055)** | 653 (678-1330), 457 (221-677), 220 (1-220), 196 (1331-1526) | 519 (211-729), 319 (774-1092), 301 (1226-1526), 210 (1-210), 133 (1093-1225), 44 (730-773) |
| *M. coragypsi* | **488 (232-719), 277 (1040-1316), 231 (1-231), 225 (815-1039), 121 (1376-1496), 95 (720-814), 59 (1317-1375)** | 442 (664-1105), 402 (233-634), 232 (1-232), 197 (1300-1496), 194 (1106-1299), 29 (635-663) | 660 (57-716), 305 (1077-1381), 136 (840-975), 115 (1382-1496), 101 (976-1076), 79 (761-839), 56 (1-56), 35 (726-760), 9 (717-725) |
| *M. cottewii* | **237 (605-841), 234 (1-234), 186 (235-420), 184 (421-604), 157 (989-1145), 147 (842-988), 105 (1146-1250), 99 (1418-1516), 85 (1251-1335), 82 (1336-1417)** | 378 (260-637), 324 (813-1136), 235 (1-235), 198 (1319-1516), 172 (1147-1318), 146 (638-783), 29 (784-812), 24 (236-259), 10 (1137-1146) | **392 (1-392), 316 (402-717), 237 (845-1081), 218 (1214-1431), 132 (1082-1213), 85 (1432-1516), 83 (762-844), 44 (718-761), 9 (393-401)** |
| *M. cricetuli* | 290 (1058-1347), 233 (385-617), 203 (855-1057), 202 (1-202), 182 (203-384), 120 (735-854), 117 (618-734), 97 (1432-1528), 84 (1348-1431) | 679 (1-679), 458 (680-1137), 198 (1331-1528), 193 (1138-1330) | 731 (1-731), 434 (1095-1528), 237 (858-1094), 82 (776-857), 44 (732-775) |
| *M. crocodyli* | **602 (1-602), 277 (1040-1316), 147 (837-983), 121 (1376-1496), 95 (720-814), 85 (635-719), 59 (1317-1375), 56 (984-1039), 32 (603-634), 22 (815-836)** | 635 (1-635), 470 (636-1105), 197 (1300-1496), 194 (1106-1299) | 607 (110-716), 305 (1077-1381), 237 (840-1076), 115 (1382-1496), 79 (761-839), 56 (1-56), 53 (57-109), 35 (726-760), 9 (717-725) |
| *M. cynos* | **488 (236-723), 276 (1046-1321), 195 (1-195), 147 (843-989), 121 (1381-1501), 95 (724-818), 59 (1322-1380), 56 (990-1045), 32 (204-235), 24 (819-842), 8 (196-203)** | **637 (668-1304), 402 (237-638), 236 (1-236), 197 (1305-1501), 29(639-667)** | 664 (57-720), 304 (1083-1386), 237 (846-1082), 115 (1387-1501), 81 (765-845), 56 (1-56), 35 (730-764), 9 (721-729) |
| *M. dispar* | **233 (383-615), 206 (1055-1260), 181 (33-213), 179 (1346-1524), 169 (214-382), 146 (853-998), 120 (733-852), 117 (616-732), 85 (1261-1345), 56 (999-1054), 32 (1-32)** | 378 (78-455), 290 (845-1134), 206 (472-677), 196 (1329-1524), 194 (1135-1328), 167 (678-844), 40 (30-69), 29 (1-29), 16 (456-471), 8 (70-77), | 519 (211-729), 318 (774-1091), 301 (1224-1524), 210 (1-210), 132 (1092-1223), 44 (730-773) |
| *M. edwardii* | **402 (235-636), 276 (1044-1319), 194 (1-194), 147 (841-987), 121 (1379-1499), 95 (723-817), 86 (637-722), 59 (1320-1378), 56 (988-1043), 40 (195-234), 23 (818-840)** | 442 (667-1108), 402 (236-637), 235 (1-235), 197 (1303-1499), 194 (1109-1302), 29 (638-666) | 663 (57-719), 304 (1081-1384), 237 (844-1080), 115 (1385-1499), 80 (764-843), 56 (1-56), 35 (729-763), 9 (720-728) |
| *M. elephantis* | **349 (368-716), 291 (1039-1329), 203 (836-1038), 176 (16-191), 176 (192-367), 120 (1389-1508), 119 (717-835)** | 679 (634-1312), 367 (74-440), 196 (1313-1508), 193 (441-633), 61 (13-73), 12 (1-12), 119 (717-835) | 658 (56-713), 319 (1076-1394), 318 (758-1075), 114 (1395-1508), 55 (1-55), 44 (714-757) |
| *M. equigenitalium* | **291 (1039-1329), 265 (368-632), 203 (836-1038), 176 (16-191), 176 (192-367), 120 (1389-1508), 119 (717-835)** | 679 (634-1312), 367 (74-440), 196 (1313-1508), 193 (441-633), 61 (13-73), 12 (1-12) | 658 (56-713), 319 (1076-1394), 318 (758-1075), 114 (1395-1508), 55 (1-55), 44 (714-757) |
| *M. equirhinis* | **354 (16-369), 349 (370-718), 293 (1040-1332), 203 (837-1039), 120 (1392-1511), 118 (719-836), 59 (1333-1391)** | 486 (636-1121), 366 (76-441), 196 (1316-1511), 194 (1122-1315), 176 (460-635), 52 (13-64), 18 (442-459), 12 (1-12), 11 (65-75) | 558 (158-715), 435 (1077-1511), 237 (840-1076), 93 (65-157), 80 (760-839), 55 (1-55), 44 (716-759), 9 (56-64) |
| *M. falconis* | **293 (1045-1337), 263 (204-466), 255 (467-721), 188 (16-203), 147 (842-988), 120 (722-841), 120 (1397-1516), 59 (1338-1396), 56 (989-1044), 15 (1-15)** | 561 (78-638), 488 (639-1126), 196 (1321-1516), 194 (1127-1320), 65 (13-77), 12 (1-12) | 518 (201-718), 435 (1082-1516), 237 (845-1081), 82 (763-844), 55 (1-55), 55 (56-110), 49 (111-159), 44 (719-762), 41 (160-200) |
| *M. fastidiosum* | **329 (274-602), 240 (603-842), 184 (1320-1503), 147 (843-989), 130 (16-145), 128 (146-273), 104 (1131-1234), 85 (1046-1130), 85 (1235-1319), 56 (990-1045), 15 (1-15)** | 493 (143-635), 479 (824-1302), 146 (636-781), 126 (1303-1428), 75 (1429-1503), 63 (13-75), 55 (76-130), 42 (782-823), 12 (1-12), 12(131-142) | 845 (1-845), 332 (1172-1503), 237 (846-1082), 89 (1083-1171) |
| *M. faucium* | **293 (1037-1329), 255 (462-716), 215 (16-230), 147 (834-980), 137 (231-367), 120 (1389-1508), 117 (717-833), 94 (368-461), 59 (1330-1388), 56 (981-1036), 15 (1-15)** | 485 (634-1118), 402 (232-633), 219 (13-231), 196 (1313-1508), 194 (1119-1312), 12 (1-12) | 556 (158-713), 435 (1074-1508), 237 (837-1073), 93 (65-157), 70 (758-827), 55 (1-55), 44 (714-757), 9 (56-64), 9 (828-836) |
| *M. felifaucium* | **352 (370-721), 292 (1044-1335), 217 (16-232), 147 (841-987), 137 (233-369), 112 (1395-1506), 95 (722-816), 59 (1336-1394), 56 (988-1043), 24 (817-840), 15 (1-15)** | 877 (234-1110), 208 (1111-1318), 188 (1319-1506), 168 (66-233), 53 (13-65), 12(1-12) | 489 (230-718), 237 (844-1080), 187 (1214-1400), 164 (66-229), 133 (1081-1213), 106 (1401-1506), 81 (763-843), 56 (1-56), 44 (719-762), 9 (57-65) |
| *M. feliminutum* | **966 (294-1259), 293 (1-293), 271 (1260-1530)** | 653 (1-653), 489 (845-1333), 197 (1334-1530), 191 (654-844) | 491 (243-733), 318 (778-1095), 242 (1-242), 234 (1096-1329), 143 (1388-1530), 58 (1330-1387), 44 (734-777) |
| *M. felis* | **285 (439-723), 279 (1046-1324), 220 (16-235), 203 (236-438), 147 (843-989), 122 (1384-1505), 95 (724-818), 59 (1325-1383), 56 (990-1045), 24 (819-842), 15 (1-15)** | 446 (668-1113), 402 (237-638), 224 (13-236), 198 (1308-1505), 194 (1114-1307), 29 (639-667), 12 (1-12) | 664 (57-720), 318 (765-1082), 307 (1083-1389), 116 (1390-1505), 56 (1-56), 44 (721-764) |
| *M. fermentans* | **382 (339-720), 350 (1043-1392), 194 (1-194), 147 (840-986), 112 (1393-1504), 107 (232-338), 95 (721-815), 56 (987-1042), 37 (195-231), 24 (816-839)** | 681 (636-1316), 232 (1-232), 212 (319-530), 188 (1317-1504), 105 (531-635), 86 (233-318) | **507 (211-717), 187 (1212-1398), 150 (843-992), 145 (66-210), 132 (1080-1211), 106 (1399-1504), 87 (993-1079), 81 (762-842), 56 (1-56), 44 (718-761), 9 (57-65)** |
| *M. flocculare* | **234 (391-624), 206 (1064-1269), 202 (862-1063), 189 (33-221), 179 (1355-1533), 169 (222-390), 120 (742-861), 117 (625-741), 85 (1270-1354), 17 (16-32), 15 (1-15)** | 471 (30-500), 290 (854-1143), 196 (1338-1533), 194 (1144-1337), 186 (501-686), 167 (687-853), 17 (13-29), 12 (1-12) | 738 (1-738), 301 (1233-1533), 236 (865-1100), 132 (1101-1232), 82 (783-864), 44 (739-782) |
| *M. gallinaceum* | **487 (236-722), 336 (1045-1380), 203 (1-203), 171 (818-988), 121 (1381-1501), 95 (723-817), 56 (989-1044), 32 (204-235),** | 473 (638-1110), 401 (237-637), 236 (1-236), 197 (1305-1501), 194 (1111-1304) | 519 (201-719), 305 (1082-1386), 237 (845-1081), 144 (57-200), 115 (1387-1501), 81 (764-844), 56 (1-56), 44 (720-763) |
| *M. gallinarum* | **816 (1-816), 291 (1044-1334), 171 (817-987), 112 (1394-1505), 59 (1335-1393), 56 (988-1043)** | 681 (637-1317), 636 (1-636), 188 (1318-1505) | 706 (57-762), 319 (1081-1399), 237 (844-1080), 106 (1400-1505), 81 (763-843), 56 (1-56) |
| *M. gallisepticum* | 535 (462-996), 227 (235-461), 192 (1053-1244), 146 (1-146), 143 (1245-1387), 122 (1388-1509), 88 (147-234), 56 (997-1052) | 401 (236-636), 212 (974-1185), 186 (788-973), 146 (642-787), 131 (1-131), 126 (1186-1311), 123 (1312-1434), 104 (132-235), 75 (1435-1509), 5 (637-641) | 937 (153-1089), 302 (1208-1509), 152 (1-152), 118 (1090-1207) |
| *M. gallopavonis* | **489 (232-720), 277 (1041-1317), 231 (1-231), 147 (838-984), 121 (1377-1497), 95 (721-815), 59 (1318-1376), 56 (985-1040), 22 (816-837)** | **636 (665-1300), 403 (233-635), 197 (1301-1497), 116 (1-116), 89 (117-205), 29 (636-664), 27 (206-232)** | 662 (56-717), 305 (1078-1382), 237 (841-1077), 115 (1383-1497), 79 (762-840), 55 (1-55), 35 (727-761), 9 (718-726) |
| *M. gateae* | **293 (1042-1334), 255 (465-719), 203 (839-1041), 201 (1-201), 169 (202-370), 119 (720-838), 96 (1418-1513), 94 (371-464), 59 (1335-1393), 24 (1394-1417)** | **487 (637-1123), 429 (208-636), 196 (1318-1513), 194 (1124-1317), 131 (77-207) , 76 (1-76)** | 558 (159-716), 435 (1079-1513), 237 (842-1078), 102 (57-158), 81 (761-841), 56 (1-56), 44 (717-760) |
| *M. genitalium* | **249 (1052-1300), 233 (819-1051), 232 (373-604), 214 (605-818), 146 (1-146), 124 (1387-1510), 95 (278-372), 89 (147-235), 59 (1328-1386), 42 (236-277), 27 (1301-1327)** | 212 (973-1184), 211 (237-447), 200 (1311-1510), 193 (448-640), 157 (816-972), 146 (641-786), 131 (1-131), 126 (1185-1310), 93 (144-236), 29 (787-815), 12 (132-143) | 592 (233-824), 304 (1207-1510), 264 (825-1088), 152 (1-152), 118 (1089-1206), 80 (153-232) |
| *M. glycophilum* | **488 (235-722), 277 (1043-1319), 194 (1-194), 147 (840-986), 121 (1379-1499), 95 (723-817), 59 (1320-1378), 56 (987-1042), 32 (203-234), 22 (818-839), 8 (195-202)** | **636 (667-1302), 402 (236-637), 197 (1303-1499), 117 (1-117), 91 (118-208), 29 (638-666), 27 (209-235)** | 663 (57-719), 305 (1080-1384), 237 (843-1079), 115 (1385-1499), 79 (764-842), 56 (1-56), 35 (729-763), 9 (720-728) |
| *M. gypis* | **352 (1040-1391), 301 (68-368), 255 (463-717), 147 (837-983), 120 (1392-1511), 119 (718-836), 94 (369-462), 67 (1-67), 56 (984-1039)** | 681 (635-1315), 429 (206-634), 196 (1316-1511), 117 (1-117), 88 (118-205) | 714 (1-714), 321 (1077-1397), 237 (840-1076), 114 (1398-1511), 81 (759-839), 44 (715-758) |
| *M. haemocanis* | 229 (577-805), 211 (276-486), 190 (1023-1212), 170 (1-170), 167 (1297-1463), 147 (820-966), 90 (487-576), 84 (1213-1296), 56 (967-1022), 46 (188-233), 42 (234-275), 17 (171-187), 14 (806-819) | 667 (613-1279), 378 (235-612), 234 (1-234), 109 (1280-1388), 75 (1389-1463) | 829 (231-1059), 288 (1176-1463), 193 (1-193), 89 (1060-1148), 37 (194-230), 27 (1149-1175) |
| *M. haemofelis* | 229 (577-805), 211 (276-486), 190 (1023-1212), 170 (1-170), 167 (1297-1463), 147 (820-966), 90 (487-576), 84 (1213-1296), 56 (967-1022), 46 (188-233), 42 (234-275), 17 (171-187), 14 (806-819) | 667 (613-1279), 378 (235-612), 234 (1-234), 109 (1280-1388), 75 (1389-1463) | 829 (231-1059), 288 (1176-1463), 193 (1-193), 89 (1060-1148), 37 (194-230), 27 (1149-1175) |
| *M. haemomuris* | **442 (1022-1463), 333 (486-818), 211 (275-485), 147 (819-965), 134 (1-134), 98 (135-232), 56 (966-1021), 42 (233-274)** | 668 (612-1279), 378 (234-611), 166 (68-233), 109 (1280-1388), 75 (1389-1463), 67 (1-67) | 1270 (194-1463), 137 (57-193), 56 (1-56) |
| *M. hominis* | **370 (1-370), 349 (371-719), 291 (1041-1331), 147 (838-984), 120 (1391-1510), 118 (720-837), 59 (1332-1390), 56 (985-1040)** | 552 (77-628), 484 (637-1120), 196 (1315-1510), 194 (1121-1314), 76 (1-76), 8 (629-636) | 558 (159-716), 433 (1078-1510), 237 (841-1077), 93 (66-158), 80 (761-840), 56 (1-56), 44 (717-760), 9 (57-65) |
| *M. hyopharyngis* | **489 (234-722), 290 (1045-1334), 193 (1-193), 118 (829-946), 110 (1394-1503), 95 (723-817), 59 (1335-1393), 56 (989-1044), 42 (947-988), 40 (194-233), 11 (818-828)** | 680 (638-1317), 403 (235-637), 234 (1-234), 186 (1318-1503) | 489 (231-719), 318 (1082-1399), 237 (845-1081), 165 (66-230), 104 (1400-1503), 81 (764-844), 56 (1-56), 44 (720-763), 9 (57-65) |
| *M. hyopneumoniae* | **233 (382-614), 206 (1054-1259), 202 (852-1053), 180 (33-212), 179 (1345-1523), 169 (213-381), 120 (732-851), 117 (615-731), 85 (1260-1344), 32 (1-32),** | 651 (677-1327), 425 (30-454), 206 (471-676), 196 (1328-1523), 29 (1-29), 16 (455-470) | 728 (1-728), 318 (773-1090), 301 (1223-1523), 132 (1091-1222), 44 (729-772) |
| *M. hyorhinis* | **291 (1048-1338), 233 (374-606), 179 (1339-1517), 169 (205-373), 147 (845-991), 133 (16-148), 121 (724-844), 117 (607-723), 56 (992-1047), 48 (149-196), 15 (1-15), 8(197-204)** | 656 (13-668), 459 (669-1127), 196 (1322-1517), 194 (1128-1321), 12 (1-12) | 720 (1-720), 433 (1085-1517), 237 (848-1084), 83 (765-847), 44 (721-764) |
| *M. hyosynoviae* | **349 (369-717), 293 (1039-1331), 231 (1-231), 147 (836-982), 137 (232-368), 120 (1391-1510), 118 (718-835), 59 (1332-1390), 56 (983-1038)** | **486 (635-1120), 402 (233-634), 196 (1315-1510), 194 (1121-1314), 156 (77-232), 76 (1-76)** | 649 (66-714), 435 (1076-1510), 237 (839-1075), 80 (759-838), 56 (1-56), 44 (715-758), 9 (57-65) |
| *M. imitans* | 535 (462-996), 227 (235-461), 192 (1053-1244), 146 (1-146), 143 (1245-1387), 122 (1388-1509), 88 (147-234), 56 (997-1052) | 401 (236-636), 212 (974-1185), 186 (788-973), 146 (642-787), 131 (1-131), 126 (1186-1311), 123 (1312-1434), 104 (132-235), 75 (1435-1509), 5 (637-641) | 937 (153-1089), 302 (1208-1509), 152 (1-152), 118 (1090-1207) |
| *M. indiense* | 349 (369-717), 293 (1039-1331), 231 (1-231), 147 (836-982), 137 (232-368), 122 (1391-1512), 118 (718-835), 59 (1332-1390), 56 (983-1038) | 486 (635-1120), 402 (233-634), 232 (1-232), 198 (1315-1512), 194 (1121-1314) | 556 (159-714), 437 (1076-1512), 237 (839-1075), 93 (66-158), 80 (759-838), 56 (1-56), 44 (715-758), 9 (57-65) |
| *M. iners* | **490 (237-726), 290 (1049-1338), 196 (1-196), 147 (846-992), 112 (1398-1509), 95 (727-821), 59 (1339-1397), 56 (993-1048), 40 (197-236), 24 (822-845)** | 680 (642-1321), 404 (238-641), 237 (1-237), 188 (1322-1509) | 490 (234-723), 318 (1086-1403), 237 (849-1085), 159 (57-215), 106 (1404-1509), 81 (768-848), 56 (1-56), 44 (724-767), 9 (216-224), 9 (225-233) |
| *M. iowae* | **719 (274-992), 249 (1136-1384), 144 (1-144), 129 (145-273), 120 (1385-1504), 87 (1049-1135), 56 (993-1048)** | 495 (142-636), 332 (783-1114), 194 (1115-1308), 146 (637-782), 121 (1309-1429), 112 (30-141), 75 (1430-1504), 29 (1-29) | 935 (151-1085), 419 (1086-1504), 150 (1-150) |
| *M. lagogenitalium* | **291 (1051-1341), 233 (378-610), 203 (848-1050), 179 (1342-1520), 169 (209-377), 151 (1-151), 120 (728-847), 117 (611-727), 57 (152-208)** | 672 (1-672), 458 (673-1130), 196 (1325-1520), 194 (1131-1324) | 519 (206-724), 433 (1088-1520), 237 (851-1087), 135 (71-205), 82 (769-850), 70 (1-70), 44 (725-768) |
| *M. leonicaptivi - leocaptivus* | **706 (16-721), 276 (1042-1317), 147 (839-985), 121 (1377-1497), 95 (722-816), 59 (1318-1376), 56 (986-1041), 22 (817-838), 15(1-15)** | 635 (666-1300), 624 (13-636), 197 (1301-1497), 29 (637-665), 12 (1-12) | 662 (57-718), 620 (763-1382), 115 (1383-1497), 56 (1-56), 35 (728-762), 9 (719-727) |
| *M. leopharyngis* | **447(373-819), 291(1047-1337), 147(844-990), 137(236-372), 115(16-130), 112(1397-1508), 73(131-203), 59(1338-1396), 56(991-1046), 32(204-235), 24(820-843), 15(1-15)** | 681 (640-1320), 403 (237-639), 224 (13-236), 188 (1321-1508), 12 (1-12) | 498 (233-730), 319 (1084-1402), 237 (847-1083), 135 (66-200), 106 (1403-1508), 81 (766-846), 56 (1-56), 35 (731-765), 32 (201-232), 9 (57-65) |
| *M. lipofaciens* | **291 (1044-1334), 266 (370-635), 200 (33-232), 147 (841-987), 137 (233-369), 112 (1394-1505), 95 (722-816), 86 (636-721), 59 (1335-1393), 56 (988-1043), 24 (817-840), 17 (16-32), 15 (1-15)** | 681 (637-1317), 403 (234-636), 204 (30-233), 188 (1318-1505), 17 (13-29), 12 (1-12) | 489 (230-718), 319 (1081-1399), 237 (844-1080), 173 (57-229), 106 (1400-1505), 81 (763-843), 56 (1-56), 44 (719-762) |
| *M. lipophilum* | **290 (1045-1334), 230 (235-464), 194 (1-194), 172 (465-636), 147 (842-988), 128 (1394-1521), 119 (723-841), 86 (637-722), 59 (1335-1393), 56 (989-1044), 32 (203-234), 8 (195-202)** | 680 (638-1317), 235 (1-235), 208 (236-443), 204 (1318-1521), 194 (444-637) | 488 (232-719), 318 (1082-1399), 237 (845-1081), 166 (66-231), 122 (1400-1521), 81 (764-844), 56 (1-56), 44 (720-763), 9 (57-65) |
| *M. maculosum* | **447 (373-819), 291 (1047-1337), 147 (844-990), 137 (236-372), 130 (1-130), 112 (1397-1508), 73 (131-203), 59 (1338-1396), 56 (991-1046), 32 (204-235), 24 (820-843)** | 681 (640-1320), 403 (237-639), 236 (1-236), 188 (1321-1508) | 489 (233-721), 319 (1084-1402), 237 (847-1083), 135 (66-200), 106 (1403-1508), 81 (766-846), 56 (1-56), 35 (731-765), 32 (201-232), 9 (57-65), 9 (722-730) |
| *M. meleagridis* | **489 (235-723), 291 (1046-1336), 202 (1-202), 171 (819-989), 112 (1396-1507), 95 (724-818), 59 (1337-1395), 56 (990-1045), 32 (203-234)** | 467 (639-1105), 403 (236-638), 235 (1-235), 214 (1106-1319), 188 (1320-1507) | 507 (214-720), 319 (1083-1401), 237 (846-1082), 157 (57-213), 106 (1402-1507), 81 (765-845), 56 (1-56), 35 (730-764), 9 (721-729) |
| *M. microti* | **328 (274-601), 244 (602-845), 148 (846-993), 144 (1-144), 144 (1242-1385), 129 (145-273), 121 (1386-1506), 105 (1137-1241), 87 (1050-1136), 56 (994-1049)** | 508 (130-637), 332 (784-1115), 194 (1116-1309), 146 (638-783), 122 (1310-1431), 75 (1432-1506), 55 (75-129), 45 (30-74), 29 (1-29) | 698 (151-848), 420 (1087-1506), 238 (849-1086), 150 (1-150) |
| *M. moatsii* | **468 (369-836), 291 (1040-1330), 180 (1331-1510), 147 (837-983), 145 (1-145), 137 (232-368), 86 (146-231), 56 (984-1039)** | **303 (817-1119), 224 (233-456), 197 (1314-1510), 194 (1120-1313), 182 (635-816), 157 (76-232), 127 (457-583), 75 (1-75), 51 (584-634)** | 758 (1-758), 434 (1077-1510), 237 (840-1076), 81 (759-839) |
| *M. mobile* | **349 (368-716), 291 (1039-1329), 177 (145-321), 144 (1-144), 120 (1389-1508), 119 (717-835), 105 (836-940), 98 (941-1038), 59 (1330-1388), 46 (322-367)** | 485 (634-1118), 318 (1-318), 194 (1119-1312), 177 (457-633), 122 (319-440), 119 (1390-1508), 77 (1313-1389), 16 (441-456) | 713 (1-713), 318 (758-1075), 160 (1208-1367), 132 (1076-1207), 114 (1395-1508), 44 (714-757), 27 (1368-1394) |
| *M. molare* | **470 (1049-1518), 233 (377-609), 203 (846-1048), 151 (1-151), 137 (240-376), 119 (727-845), 117 (610-726), 56 (152-207), 32(208-239)** | 437 (672-1108), 431 (241-671), 240 (1-240), 196 (1323-1518), 194 (1129-1322), 20 (1109-1128) | 653 (71-723), 433 (1086-1518), 237 (849-1085), 81 (768-848), 70 (1-70), 44 (724-767) |
| *M. muris* | **776 (274-1049), 249 (1137-1385), 144 (1-144), 121 (1386-1506), 87 (1050-1136), 67 (145-211), 62 (212-273)** | 445 (142-586), 187 (784-970), 146 (638-783), 145 (971-1115), 127 (1183-1309), 122 (1310-1431), 75 (1432-1506), 67 (1116-1182), 55 (75-129), 51 (587-637), 45 (30-74), 29 (1-29), 12 (130-141) | 869 (218-1086), 420 (1087-1506), 150 (1-150), 67 (151-217) |
| *M. mustelae* | **488 (234-721), 276 (1042-1317), 233 (1-233), 147 (839-985), 121 (1377-1497), 95 (722-816), 59 (1318-1376), 56 (986-1041), 22 (817-838)** | **441 (666-1106), 402 (235-636), 234 (1-234), 197 (1301-1497), 194 (1107-1300), 29 (637-665)** | 662 (57-718), 316 (763-1078), 304 (1079-1382), 115 (1383-1497), 56 (1-56), 44 (719-762) |
| *M. mycoides* ssp*. capri* | 236 (605-840), 234 (1-234), 186 (235-420), 184 (421-604), 157 (988-1144), 147 (841-987), 105 (1145-1249), 99 (1417-1515), 85 (1250-1334), 82 (1335-1416) | 378 (260-637), 352 (784-1135), 235 (1-235), 172 (1146-1317), 146 (638-783), 134 (1318-1451), 64 (1452-1515), 24 (236-259), 10 (1136-1145) | 717 (1-717), 303 (1213-1515), 237 (844-1080), 132 (1081-1212), 82 (762-843), 44 (718-761) |
| *M. mycoides* ssp*. mycoides* LC | 236 (605-840), 234 (1-234), 186 (235-420), 184 (421-604), 157 (988-1144), 147 (841-987), 105 (1145-1249), 99 (1417-1515), 85 (1250-1334), 82 (1335-1416) | 378 (260-637), 352 (784-1135), 235 (1-235), 172 (1146-1317), 146 (638-783), 134 (1318-1451), 64 (1452-1515), 24 (236-259), 10 (1136-1145) | 717 (1-717), 303 (1213-1515), 237 (844-1080), 132 (1081-1212), 82 (762-843), 44 (718-761) |
| *M. mycoides* ssp*. mycoides* SC | **370 (235-604)^a^, 236 (605-840), 234 (1-234), 186 (235-420)^b^, 184 (421-604)^b^, 157 (988-1144), 147 (841-987), 105 (1145-1249), 99 (1417-1515), 85 (1250-1334), 82 (1335-1416)** | 378 (260-637), 352 (784-1135), 235 (1-235), 172 (1146-1317), 146 (638-783), 134 (1318-1451), 64 (1452-1515), 24 (236-259), 10 (1136-1145) | **717 (1-717), 302 (1213-1514), 237 (844-1080), 132 (1081-1212), 82 (762-843), 44 (718-761)** |
| M. neurolyticum | **291 (1058-1348), 247 (1-247), 233 (385-617), 203 (855-1057), 179 (1349-1527), 137 (248-384), 120 (735-854), 117 (618-734)** | 458 (680-1137), 394 (249-642), 248 (1-248), 196 (1332-1527), 194 (1138-1331), 37 (643-679) | 731 (1-731), 433 (1095-1527), 237 (858-1094), 82 (776-857), 44 (732-775) |
| *M. opalescens* | **291 (1044-1334), 232 (1-232), 161 (475-635), 147 (841-987), 137 (233-369), 112 (1394-1505), 105 (370-474), 95 (722-816), 86 (636-721), 59 (1335-1393), 56 (988-1043), 24 (817-840)** | 681 (637-1317), 403 (234-636), 233 (1-233), 188 (1318-1505) | 489 (230-718), 237 (844-1080), 187 (1213-1399), 164 (66-229), 132 (1081-1212), 106 (1400-1505), 81 (763-843), 56 (1-56), 35 (728-762), 9 (57-65), 9 (719-727) |
| *M. orale* | 349 (369-717), 293 (1039-1331), 231 (1-231), 147 (836-982), 137 (232-368), 122 (1391-1512), 118 (718-835), 59 (1332-1390), 56 (983-1038) | 486 (635-1120), 402 (233-634), 232 (1-232), 198 (1315-1512), 194 (1121-1314) | 556 (159-714), 435 (1076-1510), 237 (839-1075), 93 (66-158), 80 (759-838), 56 (1-56), 44 (715-758), 9 (57-65) |
| *M. ovipneumoniae* | **233 (383-615), 206 (1055-1260), 202 (853-1054), 179 (1346-1524), 169 (214-382), 141 (73-213), 120 (733-852), 117 (616-732), 85 (1261-1345), 72 (1-72)** | 455 (1-455), 290 (845-1134), 222 (456-677), 196 (1329-1524), 194 (1135-1328), 167 (678-844) | 519 (211-729), 318 (774-1091), 301 (1224-1524), 210 (1-210), 132 (1092-1223), 44 (730-773) |
| *M. ovis* | **332 (510-841), 275 (1-275), 216 (276-491), 203 (842-1044), 192 (1045-1236), 167 (1322-1488), 85 (1237-1321), 18 (492-509)** | 462 (636-1097), 368 (79-446), 189 (447-635), 112 (1112-1223), 109 (1305-1413), 81 (1224-1304), 78 (1-78), 75 (1414-1488), 14(1098-1111) | 501 (259-759), 319 (854-1172), 316 (1173-1488), 160 (99-258), 98 (1-98), 94 (760-853) |
| *M. oxoniensis* | **468 (371-838), 277 (1042-1318), 233 (1-233), 147 (839-985), 137 (234-370), 121 (1378-1498), 59 (1319-1377), 56 (986-1041)** | 484 (637-1120), 402 (235-636), 197 (1302-1498), 181 (1121-1301), 118 (1-118), 89 (119-207), 27 (208-234) | 532 (231-762), 305 (1079-1383), 237 (842-1078), 174 (57-230), 115 (1384-1498), 79 (763-841), 56 (1-56) |
| *M. penetrans* | **392 (599-990), 325 (274-598), 167 (1239-1405), 144 (1-144), 129 (145-273), 98 (1406-1503), 87 (1047-1133), 84 (1155-1238), 56 (991-1046), 21 (1134-1154)** | 493 (142-634), 332 (781-1112), 194 (1113-1306), 146 (635-780), 121 (1307-1427), 112 (30-141), 76 (1428-1503), 29 (1-29) | 933 (151-1083), 420 (1084-1503), 150 (1-150) |
| *M. phocicerebrale* | **293 (1044-1336), 255 (467-721), 195 (1-195), 169 (204-372), 147 (841-987), 120 (1396-1515), 119 (722-840), 94 (373-466), 59 (1337-1395), 56 (988-1043), 8 (196-203)** | **560 (79-638), 487 (639-1125), 196 (1320-1515), 194 (1126-1319), 78 (1-78)** | 558 (161-718), 435 (1081-1515), 237 (844-1080), 104 (57-160), 81 (763-843), 56 (1-56), 44 (719-762) |
| *M. phocidae/phocae* | **293 (1044-1336), 255 (467-721), 195 (1-195), 169 (204-372), 147 (841-987), 120 (1396-1515), 119 (722-840), 94 (373-466), 59 (1337-1395), 56 (988-1043), 8(196-203)** | **509 (79-587), 487 (639-1125), 196 (1320-1515), 194 (1126-1319), 78 (1-78), 51 (588-638)** | 558 (161-718), 435 (1081-1515), 237 (844-1080), 104 (57-160), 81 (763-843), 56 (1-56), 44 (719-762) |
| *M. phocirhinis* | **489 (233-721), 291 (1044-1334), 147 (841-987), 141 (1-141), 112 (1394-1505), 95 (722-816), 91 (142-232), 59 (1335-1393), 56 (988-1043), 24 (817-840)** | 681 (637-1317), 403 (234-636), 233 (1-233), 188 (1318-1505) | 489 (230-718), 318 (763-1080), 187 (1213-1399), 155 (57-211), 132 (1081-1212), 106 (1400-1505), 56 (1-56), 44 (719-762), 9 (212-220), 9 (221-229) |
| *M. pirum* | **613 (235-847), 192 (1052-1243), 148 (848-995), 146 (1-146), 123 (1387-1509), 88 (147-234), 84 (1244-1327), 59 (1328-1386), 56 (996-1051)** | 211 (236-446), 189 (447-635), 186 (787-972), 146 (641-786), 145 (973-1117), 131 (1-131), 126 (1185-1310), 123 (1311-1433), 104 (132-235), 76 (1434-1509), 67 (1118-1184), 5(636-640) | 936 (153-1088), 303 (1207-1509), 152 (1-152), 118 (1089-1206) |
| *M. pneumoniae* | **233 (819-1051), 232 (373-604), 214 (605-818), 178 (1052-1229), 146 (1-146), 122 (1387-1508), 98 (1230-1327), 95 (278-372), 89 (147-235), 59 (1328-1386), 42 (236-277)** | 225 (237-461), 198 (1311-1508), 179 (462-640), 157 (816-972), 146 (641-786), 145 (973-1117), 131 (1-131), 126 (1185-1310), 93 (144-236), 67 (1118-1184), 29 (787-815), 12 (132-143) | 592 (233-824), 302 (1207-1508), 264 (825-1088), 152 (1-152), 118 (1089-1206), 80 (153-232) |
| *M. primatum* | **489 (234-722), 291 (1045-1335), 233 (1-233), 147 (842-988), 119 (723-841), 112 (1395-1506), 59 (1336-1394), 56 (989-1044)** | **1318 (1-1318), 188 (1319-1506)** | 489 (231-719), 319 (1082-1400), 237 (845-1081), 165 (66-230), 106 (1401-1506), 81 (764-844), 56 (1-56), 35 (729-763), 9 (57-65), 9 (720-728) |
| *M. pullorum* | **334 (982-1315), 274 (233-506), 232 (1-232), 210 (507-716), 170 (812-981), 120 (1375-1494), 95 (717-811), 59 (1316-1374)** | 667 (632-1298), 425 (207-631), 180 (1315-1494), 134 (1-134), 72 (135-206), 15 (1299-1313), 1(1314-1314) | 657 (57-713), 306 (1075-1380), 238 (837-1074), 114 (1381-1494), 79 (758-836), 56 (1-56), 44 (714-757) |
| *M. pulmonis* | **290 (1048-1337), 278 (447-724), 238 (1-238), 166 (281-446), 147 (845-991), 96 (1422-1517), 95 (725-819), 59 (1338-1396), 56 (992-1047), 42 (239-280), 25 (820-844), 25 (1397-1421)** | 484 (837-1320), 394 (240-633), 239 (1-239), 197 (1321-1517), 166 (671-836), 29 (642-670), 8 (634-641) | 665 (57-721), 237 (848-1084), 187 (1216-1402), 131 (1085-1215), 115 (1403-1517), 82 (766-847), 56 (1-56), 44 (722-765) |
| *M. putrefaciens* | **236 (605-840), 234 (1-234), 186 (235-420), 184 (421-604), 157 (988-1144), 147 (841-987), 105 (1145-1249), 99 (1417-1515), 85 (1250-1334), 82 (1335-1416)** | 378 (260-637), 323 (813-1135), 235 (1-235), 172 (1146-1317), 146 (638-783), 134 (1318-1451), 64 (1452-1515), 29 (784-812), 24 (236-259), 10(1136-1145) | **717 (1-717), 237 (844-1080), 218 (1213-1430), 132 (1081-1212), 85 (1431-1515), 82 (762-843), 44 (718-761)** |
| *M. salivarium* | **350 (372-721), 293 (1043-1335), 202 (1-202), 147 (840-986), 137 (235-371), 121 (1395-1515), 118 (722-839), 59 (1336-1394), 56 (987-1042), 32 (203-234)** | 486 (639-1124), 403 (236-638), 235 (1-235), 197 (1319-1515), 194 (1125-1318) | 519 (200-718), 436 (1080-1515), 237 (843-1079), 93 (66-158), 80 (763-842), 56 (1-56), 44 (719-762), 41 (159-199), 9 (57-65) |
| *M. simbae* | **489 (233-721), 307 (988-1294), 232 (1-232), 147 (841-987), 112 (1396-1507), 95 (722-816), 59 (1337-1395), 42 (1295-1336), 24 (817-840)** | 683 (637-1319), 403 (234-636), 233 (1-233), 188 (1320-1507), | **489 (230-718), 370 (844-1213), 155 (57-211), 110 (1292-1401), 106 (1402-1507), 81 (763-843), 78 (1214-1291), 56 (1-56), 44 (719-762), 9 (212-220), 9 (221-229)** |
| *M.* sp. bovine  group 7 | **236 (605-840), 234 (1-234), 186 (235-420), 184 (421-604), 181 (1335-1515)^a^, 157 (988-1144), 147 (841-987), 105 (1145-1249), 99 (1417-1515)^b^, 85 (1250-1334), 82 (1335-1416)^b^** | 378 (260-637), 352 (784-1135), 235 (1-235), 172 (1146-1317), 146 (638-783), 134 (1318-1451), 64 (1452-1515), 24 (236-259), 10 (1136-1145) | 717 (1-717), 303 (1213-1515), 237 (844-1080), 132 (1081-1212), 82 (762-843), 44 (718-761) |
| *M. spermatophilum* | **291 (1042-1332), 276 (444-719), 230 (1-230), 213 (231-443), 203 (839-1041), 112 (1392-1503), 95 (720-814), 59 (1333-1391), 24 (815-838)** | 487 (635-1121), 403 (232-634), 231 (1-231), 194 (1122-1315), 188 (1316-1503) | 609 (108-716), 319 (1079-1397), 237 (842-1078), 106 (1398-1503), 81 (761-841), 56 (1-56), 51 (57-107), 44 (717-760) |
| *M. spumans* | **375 (1-375), 293 (1047-1339), 255 (470-724), 147 (844-990), 120 (1399-1518), 119 (725-843), 94 (376-469), 59 (1340-1398), 56 (991-1046)** | 561 (81-641), 487 (642-1128), 196 (1323-1518), 194 (1129-1322), 80 (1-80) | 518 (204-721), 270 (1084-1353), 237 (847-1083), 165 (1354-1518), 106 (57-162), 81 (766-846), 56 (1-56), 44 (722-765), 41 (163-203) |
| *M. sturnidae* | **333 (1043-1375), 265 (370-634), 232 (1-232), 205 (635-839), 147 (840-986), 137 (233-369), 121 (1376-1496), 56 (987-1042)** | 473 (636-1108), 402 (234-635), 233 (1-233), 195 (1302-1496), 193 (1109-1301) | 417 (1080-1496), 363 (400-762), 343 (57-399), 317 (763-1079), 56 (1-56) |
| *M. sualvi* | **468 (369-836), 291 (1040-1330), 179 (1331-1509), 147 (837-983), 145 (1-145), 137 (232-368), 86 (146-231), 56 (984-1039)** | **351 (233-583), 303 (817-1119), 194 (1120-1313), 182 (635-816), 157 (76-232), 99 (1411-1509), 97 (1314-1410), 75 (1-75), 51 (584-634)** | 702 (57-758), 433 (1077-1509), 237 (840-1076), 81 (759-839), 56 (1-56) |
| *M. subdolum* | **293 (1040-1332), 255 (463-717), 231 (1-231), 147 (837-983), 137 (232-368), 120 (1392-1511), 119 (718-836), 94 (369-462), 59 (1333-1391), 56(984-1039)** | 487 (635-1121), 402 (233-634), 232 (1-232), 196 (1316-1511), 194(1122-1315) | 556 (159-714), 435 (1077-1511), 237 (840-1076), 93 (66-158), 81 (759-839), 56 (1-56), 44 (715-758), 9 (57-65) |
| *M. suis* | **333 (524-856), 289 (1-289), 277 (1060-1336), 216 (290-505), 203 (857-1059), 167 (1337-1503), 18 (506-523)** | 649 (1-649), 500 (650-1149), 109 (1320-1428), 89 (1150-1238), 81 (1239-1319), 75 (1429-1503) | 501 (273-773), 414 (774-1187), 316 (1188-1503), 272 (1-272) |
| *M. synoviae* | **371 (1-371), 277 (1044-1320), 265 (723-987), 140 (465-604), 120 (1380-1499), 118 (605-722), 93 (372-464), 59 (1321-1379), 56 (988-1043)** | 489 (815-1303), 309 (1-309), 196 (1304-1499), 194 (444-637), 177 (638-814), 134 (310-443) | 663 (57-719), 305 (1081-1385), 238 (843-1080), 114 (1386-1499), 79 (764-842), 56 (1-56), 44 (720-763) |
| *M. testudineum - cheloniae* | **371 (1-371), 277 (1044-1320), 265 (723-987), 140 (465-604), 120 (1380-1499), 118 (605-722), 93 (372-464), 59 (1321-1379), 56 (988-1043)** | 489 (815-1303), 309 (1-309), 196 (1304-1499), 194 (444-637), 177 (638-814), 134(310-443) | 660 (57-716), 300 (1226-1525), 237 (857-1093), 132 (1094-1225), 82 (775-856), 58 (717-774), 56 (1-56) |
| *M. testudinis* | **760 (235-994), 193 (1053-1245), 146 (1-146), 122 (1389-1510), 88 (147-234), 84 (1246-1329), 59 (1330-1388), 58 (995-1052)** | 228 (236-463), 186 (786-971), 171 (464-634), 146 (640-785), 135 (972-1106), 131 (1-131), 126 (1187-1312), 123 (1313-1435), 104 (132-235), 75 (1436-1510), 67 (1120-1186), 13 (1107-1119), 5 (635-639) | 937 (153-1089), 302 (1209-1510), 152 (1-152), 119 (1090-1208) |
| *M. verecundum* | **583 (237-819), 279 (1045-1323), 204 (1-204), 147 (842-988), 121 (1383-1503), 59 (1324-1382), 56 (989-1044), 32 (205-236), 22 (820-841)** | 667 (640-1306), 402 (238-639), 237 (1-237), 197 (1307-1503) | 709 (57-765), 307 (1082-1388), 237 (845-1081), 115 (1389-1503), 79 (766-844), 56 (1-56) |
| *M. wenyonii* | **332 (506-837), 271 (1-271), 216 (272-487), 192 (1041-1232), 187 (1318-1504), 147 (838-984), 85 (1233-1317), 56 (985-1040), 18 (488-505)** | 364 (79-442), 272 (822-1093), 189 (443-631), 112 (1108-1219), 102 (720-821), 95 (1410-1504), 88 (632-719), 83 (1301-1383), 81 (1220-1300), 78 (1-78), 26 (1384-1409), 8 (1094-1101), 6 (1102-1107) | 501 (255-755), 336 (1169-1504), 319 (850-1168), 254 (1-254), 94 (756-849) |
| *M. yeatsii* | **237 (605-841), 234 (1-234), 186 (235-420), 184 (421-604), 157 (989-1145), 147 (842-988), 105 (1146-1250), 99 (1418-1516), 85 (1251-1335), 82 (1336-1417)** | **378 (260-637), 324 (813-1136), 235 (1-235), 198 (1319-1516), 172 (1147-1318), 146 (638-783), 29 (784-812), 24 (236-259), 10 (1137-1146)** | **717 (1-717), 237 (845-1081), 218 (1214-1431), 132 (1082-1213), 85 (1432-1516), 83 (762-844), 44 (718-761)** |

^1^ *Mycoplasma* species with a revised taxanomy (*i.e. M. lactucae*, *M. somnilux*, *M. melaleucae*, *M. luminosum*, *M. lucivorax*, and *M. ellychniae*) are not included, while the 6 members of the *M. mycoides* cluster (*i.e. M. capricolum* sspp., *M. mycoides* sspp., and *M.* sp. bovine group 7) are.

^2^ If no restriction pattern is marked bold, other restriction enzymes (as suggested in the manuscript) are needed to differentiate this species.

^a, b^ indicate possible differences between *rrn*A (a) and *rrn*B (b)
